# Correction: Kim et al. The Suppression of Ubiquitin C-Terminal Hydrolase L1 Promotes the Transdifferentiation of Auditory Supporting Cells into Hair Cells by Regulating the mTOR Pathway. *Cells* 2024, *13*, 737

**DOI:** 10.3390/cells15080732

**Published:** 2026-04-21

**Authors:** Yeon Ju Kim, In Hye Jeong, Jung Ho Ha, Young Sun Kim, Siung Sung, Jeong Hun Jang, Yun-Hoon Choung

**Affiliations:** 1 Department of Otolaryngology, Ajou University School of Medicine, Suwon 16499, Republic of Korea; yeonju0130@naver.com (Y.J.K.); jhflyflyfly@gmail.com (J.H.H.); jhj@ajou.ac.kr (J.H.J.); 2 Department of Medical Sciences, Ajou University Graduate School of Medicine, Suwon 16499, Republic of Korea; inhee465@naver.com; 3 Department of Biomedical Sciences, Ajou University Graduate School, Suwon 16499, Republic of Korea; ylem2s1u@gmail.com

## Affiliation Corrections

In the original publication [1], there were errors regarding the affiliations of the following authors: Siung Sung and Yun-Hoon Choung. For Siung Sung, affiliation 2 has been replaced with affiliation 3: Department of Biomedical Sciences, Ajou University Graduate School, Suwon 16499, Republic of Korea. For Yun-Hoon Choung, in addition to affiliations 1 and 2, affiliation 3 has also been added.

The authors state that the scientific conclusions are unaffected. The original publication has also been updated. 
